# Development and Evaluation of Repurposed Etoricoxib Loaded Nanoemulsion for Improving Anticancer Activities against Lung Cancer Cells

**DOI:** 10.3390/ijms222413284

**Published:** 2021-12-10

**Authors:** Shadab Md, Nabil A. Alhakamy, Waleed S. Alharbi, Javed Ahmad, Rasheed A. Shaik, Ibrahim M. Ibrahim, Javed Ali

**Affiliations:** 1 Department of Pharmaceutics, Faculty of Pharmacy, King Abdulaziz University, Jeddah 21589, Saudi Arabia; nalhakamy@kau.edu.sa (N.A.A.); wsmalharbi@kau.edu.sa (W.S.A.); 2 Center of Excellence for Drug Research & Pharmaceutical Industries, King Abdulaziz University, Jeddah 21589, Saudi Arabia; 3 Mohamed Saeed Tamer Chair for Pharmaceutical Industries, King Abdulaziz University, Jeddah 21589, Saudi Arabia; 4 Department of Pharmaceutics, College of Pharmacy, Najran University, Najran 11001, Saudi Arabia; jaahmed@nu.edu.sa; 5 Department of Pharmacology & Toxicology, Faculty of Pharmacy, King Abdulaziz University, Jeddah 21589, Saudi Arabia; rashaikh1@kau.edu.sa; 6 Department of Pharmacology, Faculty of Medicine, King Abdulaziz University, Jeddah 21589, Saudi Arabia; imibrahim1@kau.edu.sa; 7 Department of Pharmaceutics, School of Pharmaceutical Education and Research, Jamia Hamdard, New Delhi 110062, India; jali@jamiahamdard.ac.in

**Keywords:** cyclooxygenase-2 inhibitors, etoricoxib, repurposing, nanoemulsion, inflammatory markers, apoptosis, lung cancer

## Abstract

In the present work, novel modality for lung cancer intervention has been explored. Primary literature has established the potential role of cyclooxygenase-2 (COX-2) inhibitor in regression of multiple forms of carcinomas. To overcome its poor water solubility and boost anticancer activity, etoricoxib (ETO) was chosen as a therapeutic candidate for repurposing and formulated into a nanoemulsion (NE). The prepared ETO loaded NE was characterized for the surface charge, droplet size, surface morphology, and in vitro release. The optimized ETO loaded NE was then investigated for its anticancer potential employing A549 lung cancer cell line via cytotoxicity, apoptotic activity, mitochondrial membrane potential activity, cell migration assay, cell cycle analysis, Caspase-3, 9, and p53 activity by ELISA and molecular biomarker analysis through RT-PCR test. The developed ETO-NE formulation showed adequate homogeneity in the droplet size distribution with polydispersity index (PDI) of (0.2 ± 0.03) and had the lowest possible droplet size (124 ± 2.91 nm) and optimal negative surface charge (−8.19 ± 1.51 mV) indicative of colloidal stability. The MTT assay results demonstrated that ETO-NE exhibited substantial anticancer activity compared to the free drug. The ETO-NE showed a substantially potent cytotoxic effect against lung cancer cells, as was evident from the commencement of apoptosis/necrotic cell death and S-phase cell cycle arrests in A549 cells. The study on these molecules through RT-PCR confirmed that ETO-NE is significantly efficacious in mitigating the abundance of IL-B, IL-6, TNF, COX-2, and NF-kB as compared to the free ETO and control group. The current study demonstrates that ETO-NE represents a feasible approach that could provide clinical benefits for lung cancer patients in the future.

## 1. Introduction

At present, mortality caused by lung cancer is leading the death rates caused by other forms of carcinomas and it has climbed up to 25% of all cancer deaths. Many people are dying because of lung cancer in contrast to that of colon, breast, and prostate cancers combined. In accordance with the reports provided by The American Cancer Society, around 235,760 new cases of lung cancer are going to be introduced in 2021 in the United States and it can cause around 131,880 deaths [1]. Therefore, researchers worldwide are devising novel modalities for the treatment and prevention of lung cancer. The lung carcinoma therapeutic regime chiefly includes radiation and chemotherapy. Both strategies have their associated pros and cons, such as resistance to anticancer medication, lack of selectivity, dose-related toxicity, and subtherapeutic concentrations in tumor tissue [2,3]. Thus, novel targets and potential formulation approaches with substantial therapeutic efficacy and minimal toxic effects are the need of the hour.

Interestingly, recent study findings have demonstrated that inflammation is a key therapeutic target for cancer intervention and that cyclooxygenase-2 (COX-2) inhibition could be a potential anticancer modality. A multitude of clinical studies have established that specific COX-2 inhibitor causes significant regression of existing carcinomas [4,5]. COX-2 is linked with carcinogenesis, neoangiogenesis, immune suppression, and prevention of apoptosis in multiple forms of cancer. However, the exact mechanisms are still not known, though it has been anticipated that it is via diminution of pro-tumoral M2 polarization of macrophages. Nonetheless, the use of COX-2 inhibitor and other NSAIDs (non-steroidal anti-inflammatory drugs) for cancer therapy remains questionable because of the associated side effects of the NSAIDs, varying from moderate gastrointestinal toxicity to cardiotoxicity [6]. Very recently, Mabrouk (2021) and associates have provided insights of the possible role of COX-2 in the progression of cancer as well as the potential of COX-2 inhibitors in the management of associated toxicities and other drawbacks. They emphasized the promising and substantial contribution of nanoemulsions in overcoming the lacunae within clinical efficacy of COX-2 inhibitors such as poor aqueous solubility and hence, scanty absorption and bioavailability [7]. Considering such studies, ETO has been selected as the drug candidate for the present study. ETO is an extensively prescribed anti-inflammatory drug that is categorized as a BCS (Biopharmaceutical Classification System) class II and demonstrates low and variable oral bioavailability attributable to its low aqueous solubility [8]. In the present research work, repurposing ETO for lung cancer intervention was investigated to decipher its therapeutic prospect, which has been duly unexplored to date.

To overcome the toxic effect and poor solubility of ETO, it was formulated as nanoemulsion (NE) to enhance its delivery at tumor sites. NEs are nanodroplet-sized and are kinetically stable colloidal isotropic systems in which two immiscible phases are homogenized to form a single phase utilizing an emulsifying agent (such as surfactant and co-surfactant) [9]. Attributable to their stability, small droplet size, and optimal solubilization properties, NEs have enormous potential for enhancing the oral delivery of poorly soluble drugs. Therefore, the current investigation aims to design ETO-NE that could deliver the drug efficiently in the dissolved form at the tumor site where it can exert its apoptotic and antiangiogenic effects. The prepared ETO-NE was characterized for the surface charge, droplet size, and surface morphology. It was also evaluated for in vitro release of ETO from the optimized NE system. The optimized NE system loaded with ETO was then investigated for its anticancer potential, employing a A549 lung cancer cells line via cytotoxicity, cell migration, apoptotic activity, mitochondrial membrane potential (MMP) activity, cell cycle analysis, Caspase-3, 9, and p53 activity by ELISA and molecular marker analysis through RT-PCR analysis.

The novelty of the present study lies in the fact that no previous work has been cited in the literature on repurposing cyclooxygenase-2 (COX-2) inhibitor etoricoxib and formulated into nanoemulsion formulation for improving anticancer activities against lung cancer cells. The concept is new and merits further research, as it holds much promise for treating lung cancer that is producing significant rises in healthcare costs on a global scale.

Recently, a study was conducted by Malviya and associates to investigate how effective self-assembled ETO containing polyelectrolyte complex stabilized cubic nanoparticles against human cancer cells. Thus far, no attempts have been made to explore the lipid nanocarrier formulation of ETO in the lung carcinoma therapeutic regime. Therefore, the present research will add valuable data for the ETO role through the NE system in lung carcinoma therapy [10].

## 2. Materials and Methods

### 2.1. Materials

ETO, MTT reagent, Transcutol HP, PEG 200, Isopropyl myristate, and Labrafac PG were procured from Sigma-Aldrich, St. Louis, MO, USA. The other important excipients, including olive oil, Tween 20, Capryol 90, and Tween 80, were obtained from Merck, Rahway, NJ, USA. Sefsol 218 and castor oil were purchased from Nikko Chemicals (Tokyo, Japan). The Caspase-3, Bax, Bcl-2 assay kit was purchased from BioVision, Milpitas, CA, USA. The Annexin V-FITC Apoptosis Detection Kit was purchased from Invitrogen Corporation, CA, USA. All the chemicals utilized in the experiments of this study were of analytical grade.

### 2.2. Formulation Design and Optimization of the Nanoemulsion System

#### 2.2.1. Solubility Study

The solubility of ETO in these excipients was thoroughly investigated when choosing an oil, surfactant, and co-surfactant for the preparation of ETO loaded NE. Briefly, excess of ETO (500 mg) was added in specified quantities (2 mL) of formulation components (such as oil, surfactant, and co-surfactant) and the mixtures were vortexed for 15 min and subsequently placed in a water bath shaker (GFL mbH D-30938, Burgwedel, Germany) for 72 h at room temperature (25 °C) [11,12]. The obtained samples were subjected to centrifugation (4000 rpm for 15 min) followed by separation of supernatant. The supernatant (0.25 mL) was solubilized in a specified volume (2.5 mL) of methanol and analyzed for ETO content with the help of UV spectrophotometry at λmax 283 nm with suitable dilution. The experiment was performed in triplicate.

#### 2.2.2. Phase Behavior Study

The selected oil and surfactant–co-surfactant mixture (S_mix_) were homogenized in various ratios such as 1:9, 2:8, 3:7, and 4:6. The oil and Smix mixtures were diligently studied for appearance of clarity and turbidity on the addition of every drop of double-distilled water. These observations provided the data (%composition of oil, Smix, and water) for constructing phase diagrams. The points at which visibly clear and transparent emulsions were obtained marked the nanoemulsification region of the pseudoternary phase diagrams. This aqueous titration was performed for different ratios (1:9, 2:8, 3:7, and 4:6) in oil and Smix mixtures and phase diagrams were plotted to select the optimized concentration of excipients for nanoemulsion formulation development [13].

### 2.3. Preparation of Etoricoxib Loaded Nanoemulsion (ETO-NE)

For preparing ETO containing NE, appropriately weighed ETO (10 mg) was dissolved in a selected concentration of oil and Smix mixture. To this mixture, a specified volume (as per the %composition of nanoemulsion mentioned in Supplementary Table S1) of double-distilled water was added with instantaneous vortexing (pulsing vortex mixer (Fisher Scientific, Hampton, NH, USA) for 5 min at room temperature (25 °C) to obtain a dispersion system with complete optical clarity [12]. The final volume of formulation was 1 mL. The obtained nanoemulsion dispersion was subjected to evaluation for thermodynamic stability studies.

### 2.4. Evaluation of ETO-NE

#### 2.4.1. Thermodynamic Stability

Various stress tests were performed on the developed ETO-NE formulation. It has heating–cooling (4 and 40 °C) and freeze–thaw (−21 and +25 °C) cycles. Next, 1 mL of the produced NE was diluted to 100 mL with double-distilled water and centrifuged (Labofuge 400 centrifuge, Thermo Scientific, MA, USA) at 5000 rpm for 30 min to investigate centrifugation stress. Any signs of phase separation or other instability (such as drug precipitation) were visually observed [11,12]. A short-term stability test was performed on selected ETO-NE for the period of 3 months by keeping the sample at room temperature (25 °C). The samples were taken at predetermined time intervals (0, 30, 60, and 90 days). The droplet size, polydispersity index and drug content were determined during storage for the stability assessment (2).

#### 2.4.2. Determination of Mean Droplet Size, Polydispersity Index (PDI), and Zeta Potential of ETO-NE

Using a Malvern zetasizer (NanoZSP, Malvern Instruments, Worcestershire, UK), the mean droplet size, PDI, and zeta potential of ETO-NE were determined using dynamic light scattering techniques. After appropriate dilution (100 times), these tests were performed at 25 °C using disposable cells and plain folded capillary cells. Double distilled water was used as diluting agent. The mean droplet size, PDI, and zeta potential were determined for the selected formulations of ETO-NE (having maximum percentage of oil concentration and passed the thermodynamic stability tests) and repeated in triplicate to observe the mean value ± SD [12]. 

#### 2.4.3. Droplet Morphology of Optimized ETO-NE

To study the droplet morphology of the optimized ETO-NE formulation (having minimum droplet size and low PDI), transmission electron microscopy (TEM) (JEOL, JEM 1010, Tokyo, Japan) was employed. The procedure followed to carry out TEM analysis was in accordance with the reported method [5]. The ETO-NE formulations (50 µL) were diluted with double-distilled water (5 mL). The negative staining of the sample was performed by placing a drop of NE onto a carbon-coated copper grid in accordance with the reported method [11,12]. The grid was allowed to air dry thoroughly and the sample was viewed through TEM. 

### 2.5. In Vitro Drug Release Study of ETO-NE

The in vitro drug release profile of prepared ETO-NE (having minimum droplet size and low PDI) was determined by adopting the dialysis bag (molecular weight 12 kDa) using paddle-type dissolution apparatus with a rotating speed set at 50 rpm [2,14]. After that, 2 mL of the ETO-NE (containing 10 mg/mL of ETO) and an aqueous suspension of ETO (containing 10 mg/mL of ETO) were filled in a dialysis bag. Phosphate buffer (pH 7.4) with a volume of 900 mL at 37 ± 2 °C was the release medium for ETO-NE. The specified volume (1 mL) of the sample was withdrawn at various time points. The sink condition was maintained by replacing it with the same volume of dissolution medium. The ETO released from developed NE system at different time points was quantified by UV–VIS spectrophotometry at λmax 283 nm [14].

### 2.6. In Vitro Cell Line Study

The lung adenocarcinoma cell lines, namely A549, were acquired from ATCC (Manassas, VA, USA). A549 cell line was cultured in RPMI 1640 Medium, which was accompanied with 10% fetal bovine serum, streptomycin, and penicillin. The cell line was incubated at 37 °C, with 5% CO_2_, under a humidified atmosphere. The cells were allowed to grow to 80–90% confluency.

#### 2.6.1. MTT Assay to Determine Cell Viability

The toxicity of ETO-NE was analyzed using the A549 cancer cell line. The cells were seeded in 96-well plates at a density of 5000 cells/well then incubated overnight. After that, the drug and formulation (ETO, ETO-NE, as well as blank NE) were added in a concentration of 0.4, 1.6, 6.3, 25, and 100 µg/mL. Subsequently, the MTT assay was performed as per the manufacturer’s manual [2,15].

#### 2.6.2. Cell Migration Assay

The wound scratch method was performed using 15 × 10^4^ cells/500 pl/well to produce the monolayer. Following trypsinization, cells were seeded on a 24-well plate followed by incubation for 24 h at 37 °C. The scratched cell monolayers were treated with different samples of ETO, ETO-NE, as well as blank NE. To picture the wound area, an inverted microscope was utilized and with the help of Image J software, the percent cell migration was calculated [16].

#### 2.6.3. Apoptotic and Cell Cycle Activity by Flow Cytometry

Annexin V staining technique was used to evaluate the apoptosis and cell cycle analysis prompted by control, ETO, ETO-NE, blank samples. Briefly, 1 × 10^5^ cells/well were seeded in 6-well plates then incubated for 24 h. The incubated wells were treated with IC_50_ concentrations of the before mentioned formulations and again subjected to incubation for 24 h at 37 °C, 5% CO_2_. Cells were washed twice with cold PBS and harvested by centrifugation before being resuspended in 100 μL of 1× binding buffer. Finally, the staining with Annexin V-FITC (10 µL) and propidium iodide (PI) solution (5 µL) was performed followed by incubation at room temperature for 20 min. The level of apoptosis and cell cycle was evaluated by the fluorescence intensity of APC annexin V measured by flow cytometry (FACS Calibur, BD Bioscience San Jose, CA, USA). The experiments were performed in independent triplicate. [2,15,17].

#### 2.6.4. Mitochondrial Membrane Potential Activity

The tetramethylrhodamine methyl ester (TMRM) assay kit was employed to evaluate MMP. Concisely, A549 cells were grown in a 96-well plate with a cell density of 1 × 10^5^ cells/well. These cultures were incubated with different formulations under investigation. The prepared samples were then washed with PBS followed by staining with TMRM at 37 °C. These preparations were incubated for 30 min in the dark. Lastly, cells were washed again with PBS to remove staining and analyzed via flow cytometer (FACS Calibur, BD Bioscience, San Jose, CA, USA) [15].

#### 2.6.5. Caspase-3, 9, and p53 Activity by ELISA

The A549 cells were seeded then incubated with different formulations. Subsequently, the samples were resuspended in ice-cold lysate buffer. The cell lysate was then incubated on ice for 10 min before being centrifuged at 10,000 g for 1 min. Throughout the trial, the manufacturer’s procedure was followed [2,15,17].

#### 2.6.6. Effect of ETO-NE on Molecular Marker Using RT-PCR

For performing RT-PCR of developed ETO-NE, complete RNA was secluded using TRI reagent (Molecular Research Center, Cincinnati, OH, USA). The cDNA was procreated from 2 μg of total RNA employing a High-Capacity cDNA Reverse Transcription Kit. The quantitative PCR was executed on a Light Cycler 480 System (Roche) using SYBR Green qPCR Master Mix following the manufacturer’s manual. The internal control implicated was β-actin. To evaluate the expression ratio of the target gene, a procedure provided by Pfaffi (2001) was followed [18].

### 2.7. Statistical Analysis

The data obtained in current investigations were presented as mean ± standard deviation (SD, *n* = 3). Student’s *t*-tests and one-way ANOVA with a Tukey post-hoc test were used to determine the statistical significance of experimental values. *p* values of less than 0.05 were considered significant.

## 3. Results and Discussion

### 3.1. Formulation Design and Optimization of the Nanoemulsion System

#### 3.1.1. Solubility Study

**Figure 1:**
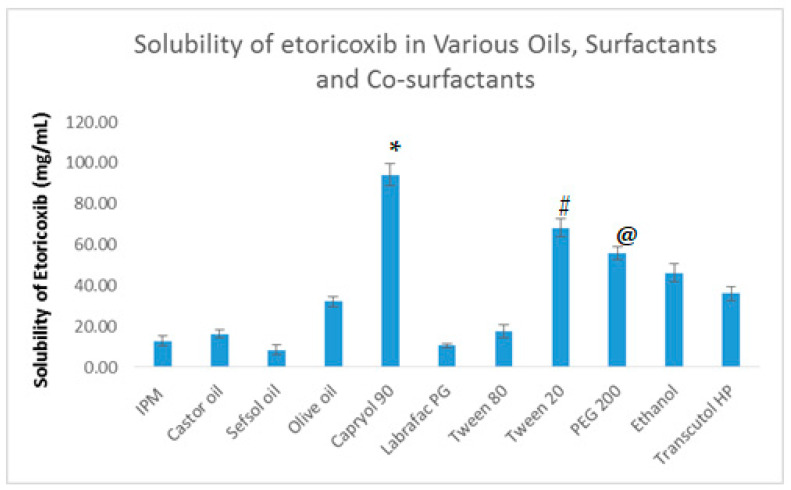
The solubility profile of ETO in different components (oils, surfactants, and co-surfactants) of the NE system. Values are expressed as mean ± SD (*n* = 3). * denotes significant difference between capryol 90 (*p* < 0.05) vs. other excipients. # denotes significant difference between tween 20 (*p* < 0.05) vs. all other excipients. @ denotes significant difference between PEG 200 (*p* < 0.05) vs. all other excipients.

The solubility of ETO was determined in various components of NE (such as oil, surfactant, and co-surfactant). It was found that ETO exhibited maximum solubility in Capryol 90 amongst oil phase; Tween 20 amongst surfactant, and PEG 200 amongst co-surfactant. The results of the solubility study in different components are shown in Figure 1. The surfactants and co-surfactant phase should also possess a desirable solubilizing capacity for ETO, but further evaluation was required to quantify the nanoemulsification efficiency for the oil phase of maximum drug solubility. Further assessment revealed that Tween 20 and PEG 200 exhibited the maximum emulsifying ability for the oil phase (Capryol 90) that had the maximum solubility of ETO in it. 

The concentration of the oil phase, Smix (Tween 20 and PEG 200) phase, and aqueous phase were finalized based on phase behavior between formulation components. It was determined exploiting the aqueous titration method. Co-surfactant (PEG 200) was completely homogenized into the chosen surfactant (Tween 20) and considered as the Smix phase in the experimental design of phase behavior study to screen the %composition of ETO-NE formulation. 

#### 3.1.2. Phase Behavior Study

**Figure 2:**
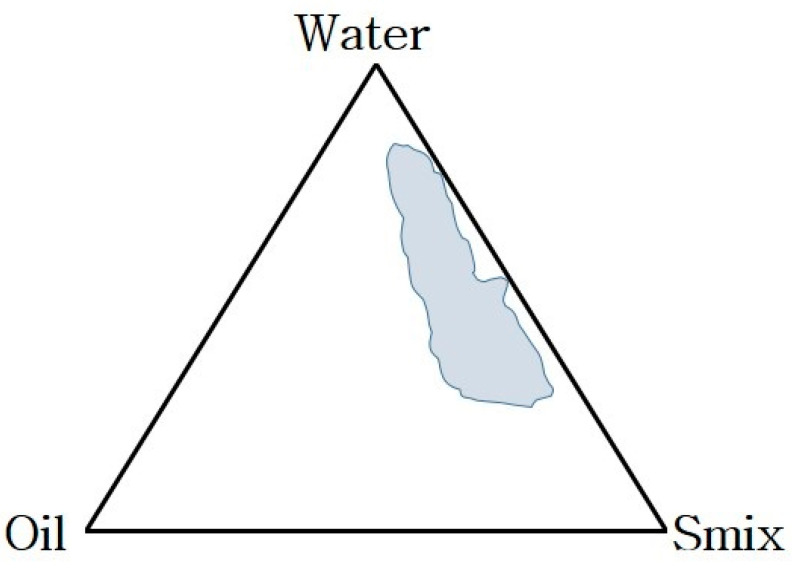
Pseudoternary phase diagram at Smix ratio 2:1 exhibiting maximum nanoemulsification region having a composition of oil phase as capryol 90 and Smix phase consist of tween 20 and PEG 200.

The different combinations of Smix ratio (1:1, 2:1, 3:1, and 4:1) and Capryol 90 influence the nanoemulsification area obtained in the phase behavior study. In the Smix phase (Tween 20 and PEG 200), when the ratio of the surfactant was double that of the co-surfactant (2:1), the nanoemulsification area obtained in the phase behavior study improved significantly (Figure 2). However, further enhancement in the Smix ratio (3:1) resulted in a decrease in the nanoemulsification area because of the liquid crystal phase appearance in the phase behavior study. All the possible %compositions of different formulations of ETO-NE that could be obtained from the phase behavior study were tested for thermodynamic stability (shown in Supplementary Table S1). However, a visibly clear, homogenous, and transparent NE appeared at a particular concentration of oil, Smix, and aqueous phase [13].

Thus, the %concentration of oil, Smix, and aqueous phase in different NE formulations of ETO were obtained through phase behavior investigation. The %composition of selected NE formulations of ETO (F1, F2, F3, and F4) from the phase diagram investigation which passed the thermodynamic stability tests are shown in Table 1.

### 3.2. Characterization of ETO-NE

The results of thermodynamic stability tests (shown in Supplementary Table S1) demonstrated that all the selected ETO-NE (F1, F2, F3, and F4) systems prepared through a low-energy emulsification technique were stable to applied stress conditions (such as heating–cooling cycles, freeze–thaw cycles, and centrifugation). The ETO-NE formulations with higher percentage of oil concentration (>17.5%) failed to sustain all the applied stress conditions and physical instability (such as coalescence and creaming) are seen (11,13). The selected ETO-NE (F1–F4) systems were found to be thermodynamically stable and therefore were subjected to further investigation. The characterization study finding revealed that the droplet size of prepared ETO–NE was significantly affected by the changes in the Smix ratio. It demonstrates that surfactant and co-surfactant substantially contribute to determining crucial physicochemical parameters such as droplet size distribution, PDI, and zeta potential of the NE (Table 2).

**Figure 3:**
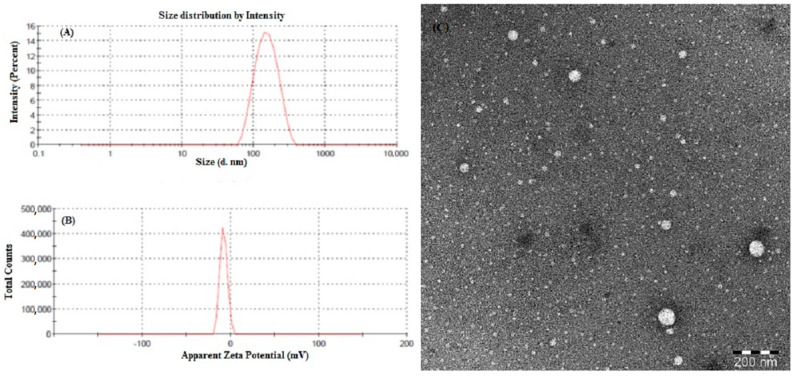
Characterization of optimized ETO-NE formulation (F2): Distribution of droplet size (**A**), and zeta potential (**B**). Droplet morphology of F2 formulation observed in Transmission Electron Microscopy (**C**).

The stability of NE is also dependent on the surface charge magnitude as the probability of coalescence between NE droplets is greatly reduced by electrical repulsive forces [13,14]. The droplet surface of the selected formulations (F1–F4) exhibited negative zeta potential (Table 2). That might be due to the occurrence of anionic groups in fatty acids and glycols in the oil phase of NE [13]. The NE formulation F2 was finally selected as an optimized formulation based on the smallest droplet size and lowest PDI (Figure 3A). Further, in vitro drug release and cancer cell line studies proceeded with optimized formulation (F2), as the results of the characterization study for optimized formulation (F2) were found to be very promising for the delivery of ETO through the NE system. The optimized NE formulation (F2) showed maximum homogeneity in the droplet size distribution with low PDI (0.24 ± 0.03) and had the smallest droplet size (124.8 ± 2.91 nm). Further, optimized NE formulation (F2) had optimal zeta potential value of −8.19 ± 1.51 mV indicating colloidal stability with minimum possibility of flocculation/coalescence or Ostwald ripening (Figure 3B) [2,13,14].

TEM image of the optimized ETO-NE (Figure 3C) showed that the droplets morphology was spherical in shape. The TEM images of the ETO-NE were in close agreement with the results obtained in zetasizer analysis. The size distribution was uniform.

The %content of ETO in optimized NE formulation (F2) was estimated by UV–visible spectrophotometer. The %content of ETO in optimized NE formulation (F2) was found to be 99.14 ± 0.33%. The short-term stability result showed small changes in droplet size, PDI and drug content with respect to time (Supplementary Figure S1). However, these changes were statistically non-significant (*p* > 0.05) at room temperature which indicated notable stability of optimized ETO-NE.

### 3.3. In Vitro Drug Release Study of ETO-NE

**Figure 4:**
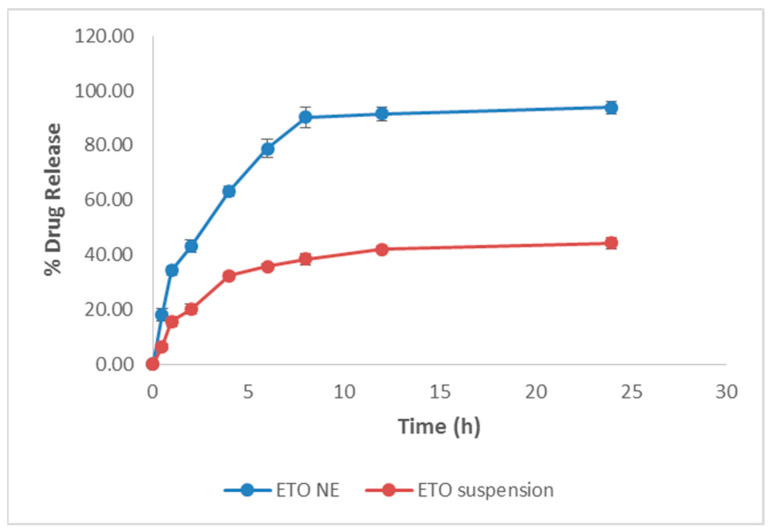
In vitro drug release profile of ETO-NE (F2) and ETO aqueous suspension in phosphate buffer of pH 7.4. Values are expressed as mean ± SD (*n* = 3). The ETO NE showed statistically significant difference (*p* < 0.001) in vitro release as compared to ETO suspension.

To get an insight into the release behavior of encapsulated ETO from optimized NE, (F2) system at the target site was carried out in phosphate buffer at pH 7.4, which corresponds to the pH of the different cellular compartments and systemic circulation. Figure 4 depicts that the release of ETO from NE in 2 h was more than 42.98 ± 2.37% whereas from aqueous suspension it was only 20.07 ± 1.96% at pH 7.4. The prepared NE exhibited the maximum release in less than 2 h, which could be attributed to droplet size in nano dimension providing a large surface area for the release of ETO from the optimized formulation system (F2). Therefore, this investigation confirmed that the optimized NE system provided an improvement in the drug release profile (*p* < 0.001) compared to the aqueous suspension system of poorly soluble drugs such as ETO (Figure 4).

### 3.4. In Vitro Cell Line Study

#### 3.4.1. Cell Viability Using MTT Assay

**Figure 5:**
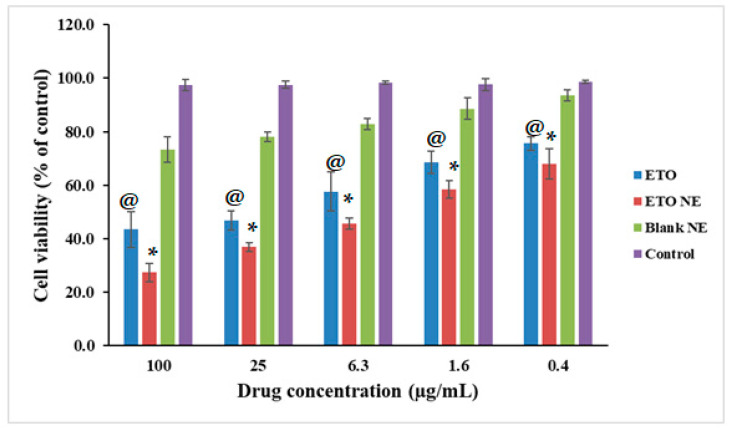
Cell viability assays of ETO NE, free ETO and blank NE group were presented. Control group is 100% cell viability. Values are expressed as mean ± SD (*n* = 3). * denotes significant difference between ETO NE (*p* < 0.05) vs. free ETO [except at 0.4 µg/mL (*p* > 0.05)] and blank NE. @ denotes significant difference between free ETO vs. blank NE (*p* < 0.05).

Figure 5 shows the cytotoxicity of the free ETO and ETO-NE against the A549 cell line. ETO and ETO-NE formulation demonstrated a concentration-dependent decrease in cell viability as shown in Figure 4. The IC_50_ value of ETO and ETO-NE against A549 was 1.29 µg/mL and 4.53 µg/mL, respectively. The results were evidence of significantly increased cytotoxicity of ETO-NE as compared to free ETO formulations (*p* < 0.005), which established the enhanced cytotoxic potential of ETO through NE formulation.

#### 3.4.2. Cell Migration Assay

**Figure 6:**
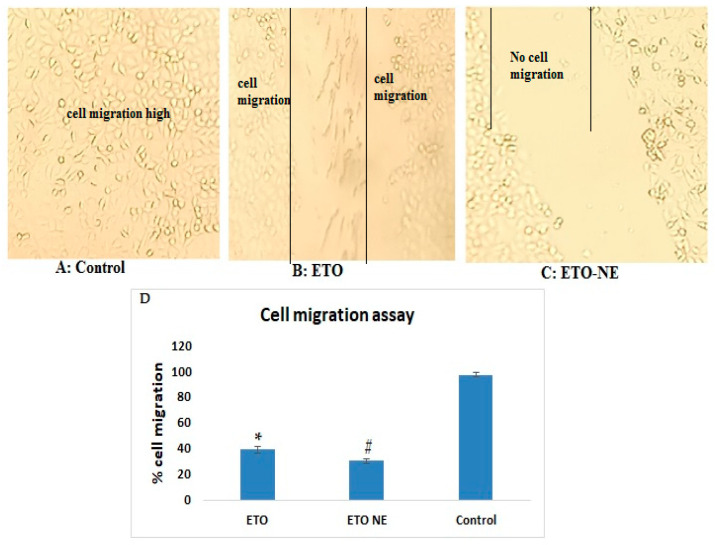
Effect of different formulations on percent cell migrated. The phase-contrast microscopy (10×) was employed to capture A549 cells migration and quantification with a scale bar of 100 µm (**A**–**C**). (**D**) Effect of different formulations on percent cell migration. Data are expressed as (mean ± SD, *n* = 3) # denotes significant difference between ETO NE (*p* < 0.05) vs. free ETO and control group. * denotes significant difference as compared to control group.

Metastasis involves the migration of cells through blood or lymph vessels resulting in the formation of distant colonies [19]. Therapeutic targeting of tumor cell invasion and migration would potentially contribute to improving the therapeutic efficacy of anticancer drugs. The extent of cells migration and proliferation can be assessed through in vitro cell migration study. The control shows a percent cell migration of 97.03% whereas ET-NE and ETO showed 39.12 ± 2.7% and 30.47 ± 1.6%. The results of the cell migration assay confirmed a significant (*p* < 0.05) reduction in cell migration for ETO-NE in comparison to control and free ETO (as shown in Figure 6). The results were indicative of potential inhibition of cell migration and thus metastasis via ETO-NE, which could lead to remarkable therapy prospects in lung carcinoma.

#### 3.4.3. Apoptotic Activity by Flow Cytometry

**Figure 7:**
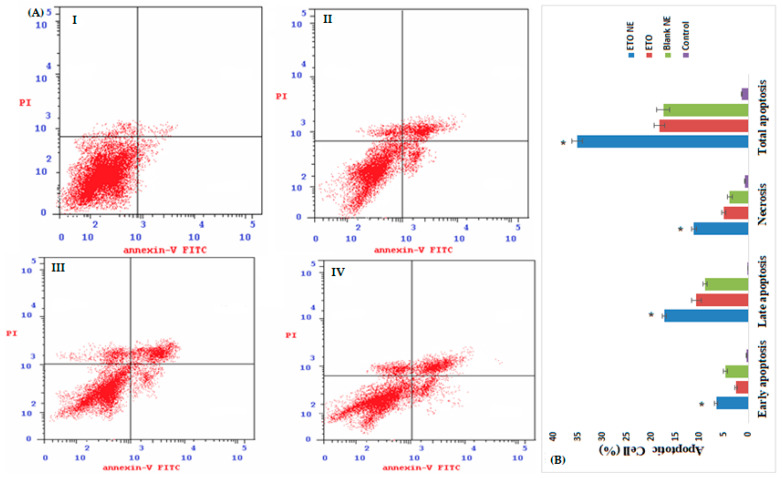
**(A**): Apoptotic and necrotic activity of A549 cells treated with (**I**) control (untreated); (**II**) blank NE; (**III**) ETO; (**IV**) ETO-NE in flow cytometry employing PI and Annexin V antibodies. (**B**) Bar graph showing early, late, and total apoptosis in A549 cells as induced by different formulations under investigation. Data are expressed as (mean ± SD, *n* = 3), * denotes significant difference between ETO NE (*p* < 0.05) vs. free ETO, blank NE and control group.

To intricately explore the cytotoxic potential of ETO-NE, apoptotic activity in the A549 cell line was evaluated by flow cytometry. The study findings revealed significant (*p* < 0.05) apoptotic cell death by ETO in comparison to the unexposed control group (Figure 7A,B). Nevertheless, when A549 cells were treated with ETO-NE, a sharp rise in apoptosis was observed, which was significantly higher (*p* < 0.05) compared to the controls and free ETO treated groups (Figure 7A). A notable increase in necrotic cell number was observed with ETO and ETO-NE compared to the control group. Furthermore, the percentage of early and late apoptotic cells treated with free ETO was significantly lower (*p* ≤ 0.05) compared with that treated with ETO-NE, suggesting a substantially increased apoptosis-inducing potential of the ETO through NE formulation (Figure 7B). Therefore, the NE formulation of ETO was shown to augment the antitumor activity of ETO utilizing the lung cancer cell line A549. It was concluded that ETO-NE was more cytotoxic than ETO naïve towards the cancerous cells; additionally, it induced distinguishing prominent apoptosis in these cells as revealed by the increased percentage of apoptotic cell death by ETO-NE as compared to both ETO naïve and the control cells.

#### 3.4.4. Cell Cycle Analysis 

**Figure 8:**
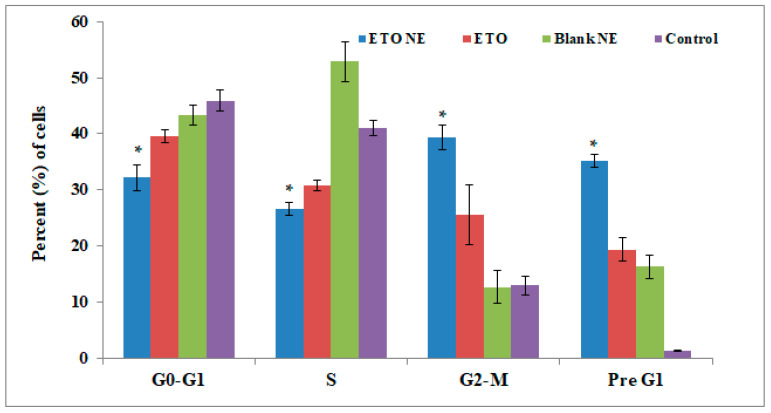
Flow cytometric analysis of ETO NE, free ETO, blank NE, and control group on cell cycle distribution in A549 cells. Data are expressed as (mean ± SD, *n* = 3), where * denotes significant difference between ETO NE (*p* < 0.05) vs. free ETO, blank NE and control group.

To find out the link between apoptosis and cell cycle arrest, PI staining followed by FACS analysis of free ETO and ETO-NE treated A549 cells was performed. Marked reduction (*p* ≤ 0.05) in the number of cells in the S phase was observed in both free ETO and ETO-NE treated groups in contrast to the control group (Figure 8). An upsurge in the percentage of apoptotic cells in the G2/M (39.3 ± 2.16%) and pre-G1 (35.15 ± 1.16%) resulted when cell cultures were treated with ETO-NE in contrast to blank and free ETO (*p* < 0.05) (Figure 8). A slight upsurge in the number of cells in the pre-G1 (19.41 ± 2.02%) phases and G2/M (25.58 ± 5.33%) was noted on treatment with free ETO compared to the blank (*p* > 0.05). Accretion of an increased number of cells in the G2/M and pre-G1 phases is a distinctive characteristic of apoptotic activity, which suggests that this increased proportion of cells would be activated for apoptosis and cell arrest via NE in the upcoming phase of the cell cycle.

In an anticancer therapeutic regime, targeting cell cycle progression could be a potential approach [2,15,17]. The outcome of the present study demonstrates that both ETO and ETO-NE effectively arrested the cell cycle at G2/M and G1/S transitions, suggestive of substantial DNA damage leading to cell death [2]. Furthermore, the reduced percentage of cells in the S-phase as a result of treatment with ETO-NE (26.28 ± 2.19%) compared to either free ETO (31.1 ± 4.33%) or control (39.68 ± 2.80%) revealed potential DNA damage through NE during the G1 phase [2]. 

#### 3.4.5. Mitochondrial Membrane Potential Activity

**Figure 9:**
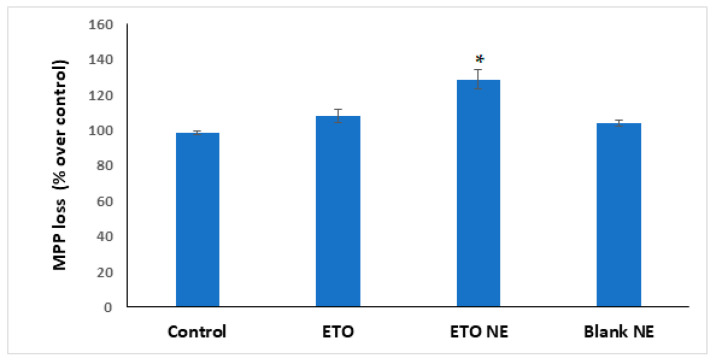
Effect of different formulations on mitochondrial membrane loss (MMP). Data are expressed as (mean ± SD, *n* = 3), where * denotes significant difference between ETO NE (*p* < 0.05) vs. free ETO, blank NE and control group.

A TMRM assay kit assessed MMP. Decreases in MMP have been associated with mitochondrial dysfunction that could lead to cell death, which denotes the anticancer ability of the chemotherapeutic agent. The results of the MMP assay demonstrated maximum MMP loss by ETO-NE as compared to free ETO and control groups (Figure 9). The results are indicative of potential A549 cell death caused by ETO-NE that marks its efficacious anticancer effect for lung cancer cells [2].

#### 3.4.6. Caspase-3, 9, and p53 Activity by ELISA Method

**Figure 10:**
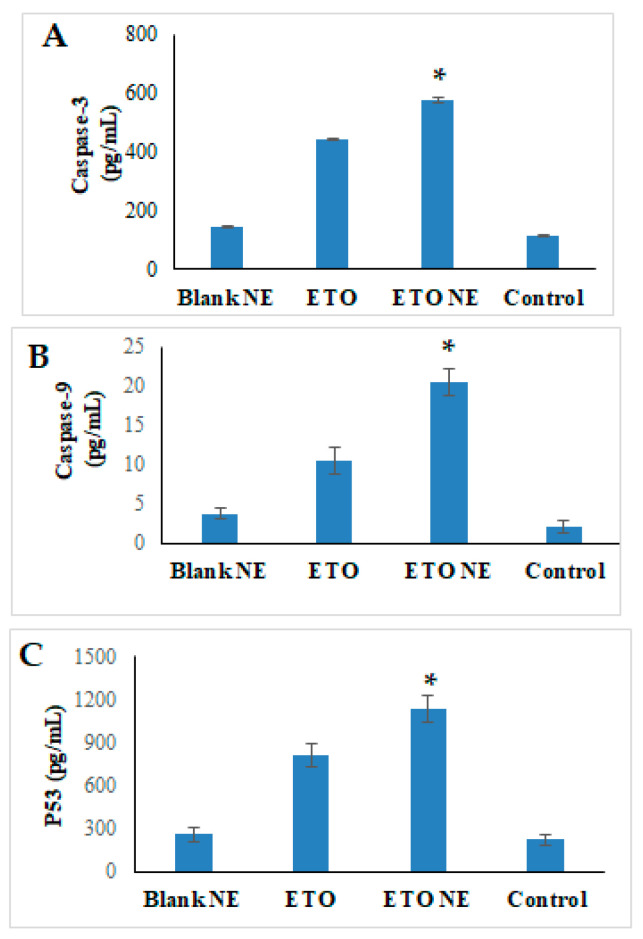
Effect of different formulation on (**A**) Caspase-3; (**B**) Caspase 9, and (**C**) p53. Data are expressed as (mean ± SD, *n* = 3) where, * denotes significant difference between ETO NE (*p* < 0.05) vs. free ETO, blank NE and control group.

Apoptosis is predominantly executed by activated caspase 3. The caspase-3 and 9 activity are closely linked with cancer cell apoptosis [2,15,17]. The present study showed that in contrast to free ETO and control groups, ETO-NE caused a significant increase (*p* < 0.05) in caspase activity (Figure 10A,B) which is attributed to the increased effectiveness of the NE resulting in enhanced effectiveness of ETO. 

Additionally, p53-mediated apoptosis is also very noteworthy in chemotherapy-associated cell death [2,15,17]. Therefore, in the current study, we performed several analyses to assess the effect of ETO-NE on p53 expression. After A549 cells were treated with ETO-NE and naïve ETO, the expression of p53 protein was increased (Figure 10C). The modifying effect of ETO-NE increased the cellular uptake of the drug as compared with the naïve ETO and NE stimulating the expression of p53 levels (Figure 10C).

#### 3.4.7. Effect of ETO-NE on Molecular Markers: Bax, Bcl-2, Nfkb, TNF, IL-B, IL-6, and COX-2 Using RT-PCR

**Figure 11:**
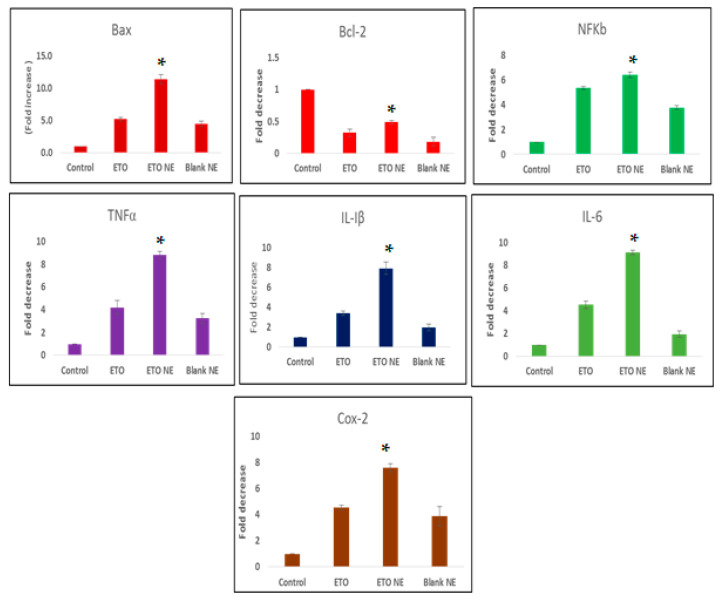
Effect of different formulations on molecular markers that play an important role in the progression of cancer. Data are expressed as (mean ± SD, *n* = 3), where * denotes significant difference between ETO NE (*p* < 0.05) vs. free ETO, blank NE and control group.

Bax protein expression plays a significant role in instigating apoptosis; however, the countenance of Bcl-2 dictates oncogenic/antiapoptotic proceeding (Figure 11) [2,20,21,22,23,24]. ETO-NE treated samples have shown a significant increment (*p* < 0.05) in Bax expression (11.40 ± 6.31 pg/mL) in contrast to that of free ETO (5.20 ± 8.04 pg/mL). It is anticipated that the reason behind such markedly improved apoptosis is the greater reach of ETO in the target cells due to increased dissolution through NE. In a similar experiment, a remarkable reduction in the expression of Bcl-2 was noticed for ETO-NE treated samples when compared with the control, which is also attributed to more accessibility of ETO in form of ETO-NE at the target site as compared to naïve ETO.

Mounting evidence confirmed the substantial role of inflammatory processes in the initiation and progression of malignant tumors. In chronic inflammation, mononuclear immune cells are filtered, apoptosis is inhibited, and angiogenesis and invasion are facilitated [25,26,27,28,29,30]. Additionally, the pro-inflammatory cytokines, such as (IL-B, IL-6, TNF), COX-2, and NF-kB are upregulated during chronic inflammation [31,32,33,34]. Collectively, a favorable niche is formed for the growth of malignant cells. Therefore, targeting this plethora of inflammatory molecules marks the therapeutic efficacy of anticancer agents [35,36]. The study on these molecules through RT-PCR confirmed that ETO-NE is significantly efficacious in mitigating the abundance of IL-B, IL-6, TNF, NF-kB, and COX-2 compared with the free ETO and control group (Figure 11). These results are evidence of the potential ability of ETO-NE in averting the progression or aggressiveness of lung cancer malignancies.

Apoptosis is very crucial for confiscating cells with severe DNA abnormalities, which otherwise might lead to aggressive carcinomas. Evading apoptosis is a well-established mechanistic for cancer therapeutics [25,30,35,36]. It was anticipated that elicitation of apoptosis in A549 cells was because of the increased cytotoxicity of the NE formulation. Both ETO and ETO-NE triggered the key apoptotic pathways that involved stimulation of Bax, a pro-apoptotic protein, and a parallel decline in Bcl-2, an antiapoptotic protein [20,25]. This improvisation of Bax/Bcl-2 expression would lead to the release of mitochondrial cytochrome C, inducing apoptosis by activation of caspases 9 and 3 [25,34,36]. This could explain a possible mechanism accountable for remarkably amplified apoptotic cell death via ETO-NE. Another possible mechanism for enhanced anticancer activity of ETO-NE is via the downregulation of the inflammatory molecules that are crucial in tumor malignancy. A similar kind of study was performed earlier and the study findings were corroborated the results found out for the present research work. Javanshir and co-workers prepared a novel nanoemulsion of Ricinus communis L. essential oil RCEO (RCEO-NE) to investigate its antioxidant and anticancer potentials. The study findings demonstrated appreciable antioxidant activities and cell-specific cytotoxic effect among the cancer HepG2 and normal L929 cells (*p* < 0.001). The study finally concluded that due to its cell-specific high-performance antioxidant, cytotoxic and individual apoptotic activities for HepG2 cancer cells, the RCEO-NE system has the potential to be applied as an efficient cancer therapy strategy [37]. Furthermore, very recently Alhakamy and associates prepared ETO loaded nanoemulsion using essential oil (eucalyptus oil). Their study findings encourage establishing a successful NE-gel formulation of ETO for analgesic and anti-inflammatory activity [14].

## 4. Conclusions

To date, ETO has never been investigated for lung cancer intervention through NE formulation. The developed ETO-NE significantly increased the dissolution and accessibility of ETO at the tumor site, as is evident from in vitro release and cell line studies. Importantly, the potential cytotoxicity of ETO-NE on A549 lung cancer cell line was established via MTT assay for cytotoxicity, apoptotic activity, MMP activity, cell cycle analysis, caspase-3, -9, and p53 activity by ELISA, and molecular marker analysis through RT-PCR. The ETO-NE was proven very efficacious in prompting pro-apoptotic Bax, whereas it mitigated antiapoptotic Bcl-2 expression in contrast to free ETO or control NEs. The results of cycle arrest analysis confirmed that ETO-NE significantly inhibited DNA synthesis as evidenced by reduced percentage of cells in the S phase in comparison to free ETO. The present study findings on inflammatory molecules through RT-PCR confirmed that ETO-NE is significantly efficacious in mitigating the abundancy of IL-B, IL-6, TNF, NF-kB, and COX-2 compared to free ETO and control groups. The promising chemotherapeutic agent, ETO-NE, for lung cancer intervention has emerged and been brought to light from the study findings of the present research work, which need further preclinical and clinical translation.

## Figures and Tables

**Table 1:** Composition of selected ETO-NE passed thermodynamic stability tests.

Formulation Code	Smix Ratio	Oil (%)	Surfactant (%)	Co-Surfactant (%)	Water (%)
F1	1:1	17.5	26.25	26.25	30.0
F2	2:1	17.5	35.0	17.5	30.0
F3	3:1	17.5	39.37	13.13	30.0
F4	4:1	17.5	42.0	10.5	30.0

**Table 2:** Characterization of selected ETO-NE.

Formulation Code	Droplet Size ± SD	PDI ± SD	Zeta Potential ± SD
F1	154.1 ± 9.2 ^a^	0.34 ± 0.04 ^a^	−4.78 ± 0.41 ^a^
F2	124.8 ± 2.9 ^b^	0.24 ± 0.03 ^b^	−8.19 ± 1.51 ^b^
F3	178.3 ± 5.4 ^c^	0.38 ± 0.07 ^c^	−13.60 ± 0.80 ^c^
F4	272.0 ± 15.4 ^d^	0.50 ± 0.06 ^d^	−14.94 ± 1.73 ^d^

Value denotes Mean ± SD; ^a–d^ samples are statistically significant (*p* < 0.05) from each other except at zeta potential value, where ^c^ and ^d^ are non-significant (*p* > 0.05).

## Data Availability

The data presented in this study are available in article.

## Supplementary Materials

The following are available online at https://www.mdpi.com/article/10.3390/ijms222413284/s1.
