# Supplementary material for: Development and Evaluation of Repurposed Etoricoxib Loaded Nanoemulsion for Improving Anticancer Activities against Lung Cancer Cells
Source: Int J Mol Sci. 2021 Dec 10;22(24):13284. doi: 10.3390/ijms222413284 (PMC8705699; doi:10.3390/ijms222413284)
Supplement: Supplementary file 1 [file ijms-22-13284-s001.zip › ijms-1472895-supplementary.pdf]

Table S1: Percentage composition of ETO-NE obtained from phase behavior study and their thermodynamic stability. (√) indicates that formulation passes the thermodynamic stability test; (x) indicates that formulation not passes the thermodynamic stability test

**Formulation**	**%Oil**	**%Surfactant**	**%co-surfactant**	**%Water**	**Thermodynamic stability**		
					Heating cooling	*Centrifugation*	*Freeze-thaw*
NE$_1$	15.0	27.5	27.5	30.0	√	√	√
NE$_2$	15.0	36.66	18.33	30.0	√	√	√
NE$_3$	15.0	41.25	13.75	30.0	√	√	√
NE$_4$	15.0	44.0	11.0	30.0	√	√	√
NE$_5$	17.5	26.25	26.25	30.0	√	√	√
NE$_6$	17.5	35.0	17.5	30.0	√	√	√
NE$_7$	17.5	39.37	13.13	30.0	√	√	√
NE$_8$	17.5	42.0	10.5	30.0	√	√	√
NE$_9$	20.0	25.0	25.0	30.0	√	√	x
NE$_{10}$	20.0	33.34	16.66	30.0	√	√	x

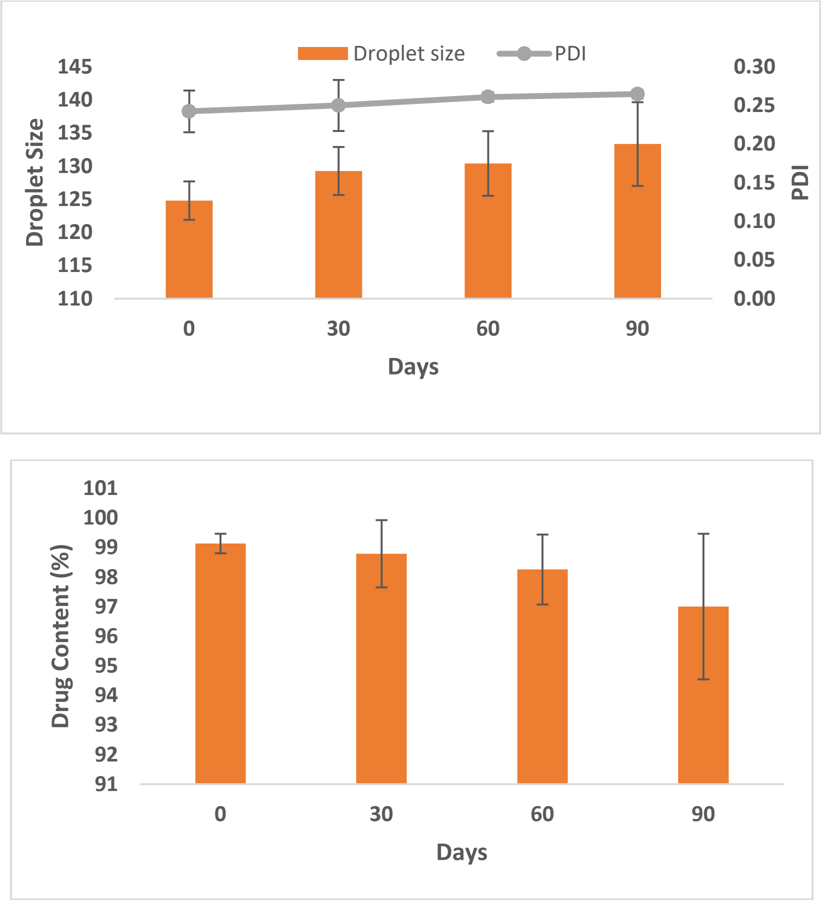

Figure S1: Short term stability study of optimized ETO NE at 25°C
